# Retroviral analysis reveals the ancient origin of Kihnu native sheep in Estonia: implications for breed conservation

**DOI:** 10.1038/s41598-020-74415-z

**Published:** 2020-10-15

**Authors:** Eve Rannamäe, Urmas Saarma, Anneli Ärmpalu-Idvand, Matthew D. Teasdale, Camilla Speller

**Affiliations:** 1 BioArCh, Department of Archaeology, University of York, York, UK; 2 Department of Archaeology, Institute of History and Archaeology, University of Tartu, Tartu, Estonia; 3 School of History, Culture and Arts Studies, University of Turku, Turku, Finland; 4 Department of Zoology, Institute of Ecology and Earth Sciences, University of Tartu, Tartu, Estonia; 5 Kihnu Native Sheep Society, Pärnu, Estonia; 6 Department of Archaeology, McDonald Institute for Archaeological Research, University of Cambridge, Cambridge, UK; 7 Department of Anthropology, University of British Columbia, Vancouver, BC Canada

**Keywords:** Genetics, Zoology, Environmental social sciences

## Abstract

Native animal breeds constitute an invaluable pool of genetic resources in a changing environment. Discovering native breeds and safeguarding their genetic diversity through specific conservation programs is therefore of high importance. Endogenous retroviruses have proved to be a reliable genetic marker for studying the demographic history of sheep (*Ovis aries*). Previous research has revealed two migratory episodes of domesticated sheep from the Middle East to Europe. The first episode included predominantly ‘primitive populations’, while the second and most recent is hypothesised to have included sheep with markedly improved wool production. To examine whether the recently discovered Kihnu native sheep in Estonia have historically been part of the first migratory episode and to what extent they have preserved primitive genetic characters, we analysed retroviral insertions in 80 modern Kihnu sheep and 83 ancient sheep from the Bronze Age to Modern Period (850 BCE–1950 CE). We identified that the Kihnu sheep have preserved ‘primitive’, ‘Nordic’, and other ‘ancient’ retrotypes that were present both in archaeological and modern samples, confirming their shared ancestry and suggesting that contemporary Kihnu native sheep originate from the first migratory episode. However, over the course of history, there has been a gradual decrease in the frequency of primitive retrotypes. Furthermore, Kihnu sheep possessed several ‘novel’ retrotypes that were absent in archaeological individuals, but were shared with improvement breeds, suggesting recent crossing within the last two centuries. To preserve these ancient lineages, our results are being applied in the conservation program of the Kihnu Native Sheep Society.

## Introduction

Conservation of native breeds is a highly important component for preserving biological diversity and cultural heritage. The recently discovered Kihnu native sheep, which have been developed in the particular climatic and geographical conditions of Estonia over thousands of years, are on the verge of extinction. Without a science-based conservation program and an expansion in numbers, Estonia will lose a crucial part of its genetic resource heritage. Furthermore, the native Kihnu sheep has been instrumental in the development of Estonia’s coastal cultural landscape, which has exceptional biodiversity value, especially in terms of its semi-natural habitats. Kihnu cultural space consists of Kihnu and neighbouring small island Manija. Its culture, communal lifestyle, local dialect, traditions, and music are what make it unique. Local handicraft and native traditions are interwoven with the usage of the wool of the indigenous Kihnu sheep. Kihnu Cultural Space was included in UNESCO’s list of Masterpieces of Oral and Intangible Heritage of Humanity in 2003, and was inscribed on the Representative List of the Intangible Cultural Heritage of Humanity in 2008.

### Brief history of the local sheep breeds in Estonia

In Estonia, the first evidence of domestic animals, including sheep (*Ovis aries*), comes from the Late Neolithic around 5000 years ago. Animal husbandry as means for subsistence, however, became important only around 2000 years later, during the Late Bronze Age^1^, evidenced by a sudden increase in faunal remains at archaeological sites from that period (e.g., Asva and Ridala settlements, Saaremaa Island). From the Late Bronze Age until the beginning of the twentieth century, zooarchaeological evidence displays a relative stability in sheep exploitation, predominantly as a source of wool and meat. This continuity in sheep husbandry has been further supported by genetic evidence: several mitochondrial DNA haplotypes have been consistently present since the Late Bronze Age as well as in modern individuals of a current native breed—the Kihnu native sheep^2, 3^.

Today, there are three local sheep breeds in Estonia: Estonian Blackhead, Estonian Whitehead, and Kihnu native sheep. The first two were developed in the 1920s and by 1958 achieved their official breed status: Blackhead was developed by crossing local indigenous sheep with the Shropshire and Oxford Down and later improved with German Blackface, Latvian Darkhead, and Suffolk; Whitehead was developed by crossing local white-faced coarse wool ewes with English Leicester and Cheviot rams and later improved with Ile-de-France, Finnsheep, Texel, Norwegian Dala, and Dorset^4^. The third breed—Kihnu native sheep—represents the locally adapted indigenous population. This breed was established based on the remaining flocks discovered in the 1990s on the Kihnu and Manija Islands, in south-western Estonia, representing peripheral populations that had not been affected to a large extent by the breeding programs of the twentieth century. In 2016, the Kihnu native sheep breed was officially acknowledged, and in 2019, it was enlisted as an endangered breed by the Ministry of Rural Affairs of Estonia. Other populations of indigenous sheep elsewhere in Estonia have not been incorporated into breeding programs and their current status is unknown.

### Position of the Kihnu native sheep in the Northern Short-tailed (NST) group

Despite their recent acknowledgement, the position of the Kihnu native sheep among the rest of the northern sheep population, namely the Northern Short-tailed (NST) group of breeds, is still under discussion. The NST group represents “an ancient type of sheep that continues to exhibit primitive characteristics” including a short tail, small size, and range of colours^4^. In Kihnu sheep, these characteristics, together with their reproductivity, behaviour, and known population history, indicate that they belong to this group as well, although the tail length has been debated (see Supplementary Text S2.2.2 online). In *Mason’s World Encyclopedia of Livestock Breeds and Breeding*^4^, Estonian native sheep have indeed been considered as part of the group, but note that the encyclopaedia was published before the acknowledgement of the Kihnu native breed, and thus mentions only the different local varieties with no breed organization.

Until now, genetic analyses of microsatellite markers and mitochondrial DNA have indicated that the Kihnu sheep (and other previously studied flocks of indigenous sheep in Estonia) are genetically distinct, divergent from modern breeds, and affiliated to ancient populations^2, 3, 5–7^. While additional morphometric and morphological studies are currently underway (including analysis of tail length, for preliminary observations see Supplementary Text S2.2.2 online), more genetic analyses are needed as well to clarify the origin and breeding history of the Kihnu native sheep. In this paper, we contribute to this exploration through the examination of endogenous retroviruses.

### Endogenous Jaagsiekte sheep retroviruses (enJSRVs)

Endogenous retroviruses (ERVs) or proviruses are "remnants of ancient retroviral infections of the host germline transmitted vertically from generation to generation" ^8^. In the sheep genome, at least 32 copies of ERVs have been described, and since they are highly related to still active exogenous betaretrovirus Jaagsiekte sheep retrovirus (JSRV), they have been termed as enJSRVs^8–11^. enJSRVs can be divided into ‘ancient’ (present in all sheep) and ‘modern’ (present in different populations) and have proved to be a good marker for studying the origins and population history of sheep^8, 12^. Among these studies, the most central example for the current study was that published by Chessa et al.^13^, where they showed two major migratory episodes of domestic sheep based on retrovirus integrations: the first migration out of the Near East, the genetic signature of which has been preserved the ‘primitive’ groups in the peripheries—the feral mouflon and northern short-tailed sheep; and later, a second migration of improved wool sheep (possibly also out of Near East), which laid the foundation for today’s ‘modern’ breeds. For the last decade, other scholars have used the same approach using enJSRVs in phylogeographic and population genetics studies, mainly to identify and preserve primitive breeds with rare gene pools^14–19^. Recently, first attempts have been made to apply this method to ancient sheep samples^20^.

### Aims and importance of the study

In this paper, we analyse retrovirus integrations to explore the population history of the native sheep in Estonia. We provide a novel extension to this method by analysing the present day Kihnu native sheep (n = 80) together with ancient populations (n = 83) from Estonian archaeological sites dating from the Late Bronze Age to Modern Period (850 BCE–1950 CE). We demonstrate that combining the results from both modern and ancient DNA can provide a comprehensive overview of the development of sheep populations through time. By focusing on a single country and one local sheep breed, we demonstrate how studying 3000 years of continuous sheep husbandry can address a gap in the overall knowledge of the NST sheep group and contribute to the wider understanding of the development and preservation of these northern populations.

## Methods

### Modern and archaeological sheep samples

Our analysis was based on three sets of samples: (1) ancient sheep remains from Estonian archaeological sites, (2) modern Kihnu native sheep, and (3) previously published reference data for modern native and other breeds. We were aware of the unequal and occasionally small sample size of archaeological specimens (especially from the Late Bronze Age) and some of the reference breeds, which derived from (a) contextual, ethical, and DNA preservation related circumstances of using ancient faunal remains and (b) availability of previously published data. The sample size of the Kihnu native sheep we regard as comprehensive, although it is still only a selection of the whole population. All restrictions deriving from sample size were considered during data interpretation.

Ancient specimens tested for enJSRVs (n = 83) were recovered from the Late Bronze Age to Modern Period (850 BCE–1950 CE). Of these, DNA from 70 had been previously extracted^2, 3^ with DNA from an additional 13 samples extracted for this study. Due to the osteological similarities between sheep and goat (*Capra hircus*), the species identities for five bone samples had to be confirmed prior to DNA extraction using collagen peptide mass fingerprinting (also known as ZooMS—Zooarchaeology by Mass Spectrometry)^21^. All five samples were confirmed to be sheep (*Ovis* spp.) (Supplementary Text S2.1.4 online). Additionally, six samples were radiocarbon dated by AMS (Supplementary Table S1-1, Text S2.1.5 online). Ancient specimens were allocated into four periods: Late Bronze Age 850–500 BCE (n = 7), Iron Age 500 BCE–1250 CE (n = 27), Middle Ages 1250–1550 CE (n = 28), and Modern Period 1550–1950 CE (n = 21). All pre-PCR lab work was done in a lab dedicated to ancient DNA and all post-PCR work was done separately (see Supplementary Text S2.1.3 online).

The blood samples of the Kihnu native sheep (n = 80; representing roughly 17% of the current breeding population of 480 animals) had been collected between 2007 and 2017. Selected samples were as diverse as possible: from different maternal lineages and with as many diverse and indigenous phenotypes as possible. The familial origins of the selected individuals were confirmed, and thus, only the most distantly related individuals were selected (for lab protocol, see Supplementary Text S2.1.2 online).

For comparison, we used previously published enJSRV data by Chessa et al.^13^. We selected only those breeds who belong to the NST group (in total 17 breeds, n = 229) and those used for improvement in the twentieth century in Estonia (in total four breeds, n = 94).

All experiments were performed in accordance with relevant guidelines and regulations and all experimental protocols were approved by the University of Tartu (Estonia) and University of York (United Kingdom), where the work was carried out. For ancient archaeological samples, the sampling permits were given by the holding institutions (see Supplementary Table S1-1 online); for modern samples, no permits or ethical approval was required, since the blood samples were collected for a routine veterinary care by veterinarian A. Ä.-I.. All sample data, reference dataset, and lab protocols can be found in Supplementary Information online.

### Data analysis

As in Chessa et al.^13^, we also focused on four ‘modern’ independently inherited insertionally polymorphic enJSRVs, which have shown to be the most informative for analysing the history of domestic sheep—enJSRV-7, enJSRV-18, enJS5F16, and enJSRV-8. We tested for the presence/absence of each of those enJSRVs through the polymerase chain reaction (PCR) (for primers and PCR conditions, see Supplementary Text S2.1.6 online). Based on the combination of the presence/absence of the four enJSRVs, we assigned individuals into so-called ‘retrotypes’ (Supplementary Table S1-4 online)^13^. To estimate the genetic relationship between the populations, we compared the frequency of each enJSRV and retrotype between the Estonian ancient populations, Kihnu native sheep, and reference data. To test for significant differences in provirus frequencies and retrotype distribution between Estonian ancient and modern populations, we conducted a Pearson’s chi-square test^22^ (for the used numerical values, see Table 1, ‘Ancient—total’ and ‘Kihnu’).

**Table 1:** Presence of four main retroviruses in ancient and modern sheep populations in Estonia, compared to the reference populations by Chessa et al.^13^. Shown are the number and proportion of individuals with the provirus insertion in each population.

No on Fig. 1	Time period/breed	Country*	Group*	Number of individuals				Frequency (%)				Total no of ind.
				enJSRV-7	enJSRV-18	enJS5F16	enJSRV-8	enJSRV-7	enJSRV-18	enJS5F16	enJSRV-8	
	Ancient—Late Bronze Age	EST	NST	3	1	3	0	60	20	60	0	5
	Ancient—Iron Age	EST	NST	6	5	7	0	38	31	44	0	16
	Ancient—Middle Ages	EST	NST	1	9	7	0	7	60	47	0	15
	Ancient—Modern Period	EST	NST	1	13	8	0	6	76	47	0	17
**1**	**Ancient—total**	**EST**	**NST**	**11**	**28**	**25**	**0**	**21**	**53**	**47**	**0**	**53**
**2**	**Kihnu**	**EST**	**NST**	**11**	**60**	**29**	**24**	**14**	**75**	**36**	**30**	**80**
3	Viena sheep	RUS	NST	2	5	4	0	40	100	80	0	5
4	Romanov	RUS	NST	2	8	10	0	20	80	100	0	10
5	Udmurtia	RUS	NST	3	5	0	0	60	100	0	0	5
6	Finnsheep	FIN	NST	0	10	11	1	0	59	65	6	17
7	Kainuu Grey	FIN	NST	0	5	10	0	0	33	67	0	15
8	Aland	FIN	NST	1	7	8	0	7	47	53	0	15
9	Gute	SWE	NST	0	5	0	2	0	45	0	18	11
10	Gotland	SWE	NST	0	11	1	0	0	73	7	0	15
11	Rya	SWE	NST	4	5	2	0	27	33	13	0	15
12	Feral sheep	NOR	NST	6	10	9	0	40	67	60	0	15
13	Old Spael	NOR	NST	1	6	4	0	17	100	67	0	6
14	Icelandic	ISL	NST	6	7	15	0	29	33	71	0	21
15	Icelandic leader	ISL	NST	5	1	12	0	33	7	80	0	15
16	Faroe	FRO	NST	2	8	9	2	20	80	90	20	10
17	Soay	GBR	NST	26	6	21	0	74	17	60	0	35
18	Hebridean	GBR	NST	4	2	5	0	67	33	83	0	6
19	Orkney	GBR	NST	0	0	1	0	0	0	8	0	13
20	Leicester	GBR	Impr	13	21	28	8	45	72	97	28	29
21	Dorset	GBR	Impr	1	16	12	0	6	100	75	0	16
22	Suffolk	GBR	Impr	1	15	8	0	7	100	53	0	15
23	Texel	NLD	Impr	13	34	32	12	38	100	94	35	34
Total:				112	275	256	49	25	60	56	11	456

*EST Estonia, *RUS* Russia, *FIN* Finland, *SWE* Sweden, *NOR* Norway, *ISL* Iceland, *FRO* Faroe Islands, *GBR* United Kingdom, *NLD* Netherlands, *NST* Northern Short-tailed, *Impr.* improvement breed.

**Figure 1:**
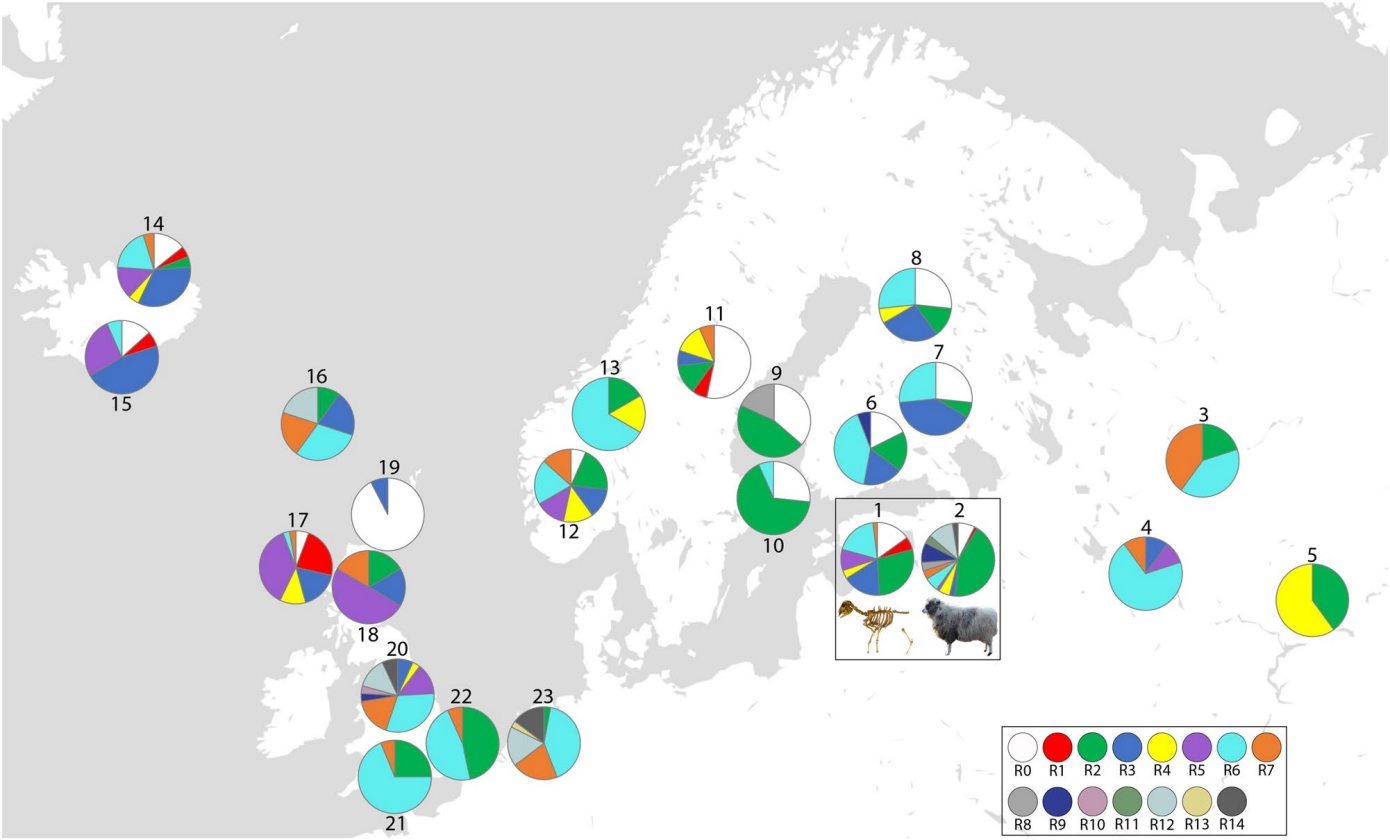
Distribution of retrotypes, where 1 and 2 are sheep populations analysed in this study and 3–23 are selected reference populations from Chessa et al.^13^. Breeds and number of individuals: 1—ancient Estonia (53); 2—Kihnu native sheep (80); 3—Viena (5); 4—Romanov (10); 5—Udmurtia (5); 6—Finnsheep (17); 7—Kainuu Grey (15); 8—Aland (15); 9—Gute (11); 10—Gotland (15); 11—Rya (15); 12—Feral (15); 13—Old Spael (6); 14—Icelandic (21); 15—Icelandic leader (15); 16—Faroe (10); 17—Soay (35); 18—Hebridean (6); 19—Orkney (13); 20—Leicester (29); 21—Dorset (16); 22—Suffolk (15); 23—Texel (34). The Texel is positioned geographically in The Netherlands, where this breed originates, although the samples had been collected from flocks in UK, USA, and Denmark^13^. For details of retrotype distribution, see Supplementary Table S1-6 online. Map is recreated based on Chessa et al. Fig. 2^13^. Map template: Snazzy Maps (CC 0). Image processing: Adobe Illustrator CS5 v.15^29^.

## Results

### Presence/absence of the enJSRVs

The results of this study rest on a presumption that the obtained data is authentic, ascertained by negative controls in DNA extraction and in PCRs and the performance of multiple PCR reactions (see Supplementary Text S2.1.7 online). Only samples with high-confident results were selected for the analysis. All 80 Kihnu sheep samples were successfully amplified and were included in the final analyses. Among ancient samples, 53 out of 83 had sufficient DNA preserved (success rate 64%) to provide confident results in the amplification of the provirus loci in individuals from all time periods: from the Late Bronze Age (n = 5, success rate 71%), Iron Age (n = 16; 59%), Middle Ages (n = 15; 54%), and Modern Period (n = 17; 81%).

To evaluate genetic relatedness, we compared frequencies of each provirus in the analysed groupings (Table 1). Most informative were the frequencies of enJSRV-7, enJSRV-18, and enJSRV-8; the former is indicative of primitive breeds, while the latter two are suggestive of recent breed improvement^13^.

**enJSRV-7** is the oldest provirus and most frequent in the most primitive populations like the Mediterranean Mouflon and Soay^13^. It was also the most frequent in the earliest tested samples from Estonia—in the Late Bronze Age and to lesser extent in the Iron Age. From the Middle Ages, however, we noted a substantial decline in the presence of enJSRV-7, which continued into the Modern Period and also into the Kihnu sheep, where this provirus had the lowest frequency (14%) compared to the other tested proviruses.

The presence of **enJSRV-18** indicates a more recent ancestry^13^. Among the modern Kihnu samples, enJSRV-18 had the highest frequency (75%), which coincides with its wide distribution in most of the other analysed reference populations, except in some of the NST breeds. Interestingly, a gradual increase was seen in the frequency of enJSRV-18 from the Late Bronze Age to the Modern Period and into the Kihnu native sheep. This suggests that the presence of enJSRV-18 in Estonia has increased in time, especially with the Middle Ages and Modern Period, when its frequency more than doubled. According to Chessa et al.^13^, the higher frequency of enJSRV-18 characterizes the wool sheep from the second migration wave. The higher frequency in Estonian populations could reflect the introduction of improved lineages which coincides with the expansion of the medieval trading network and the beginning of early modern breed improvement. A local selection that favoured enJSRV-18 and wool quality is also a possibility. However, as Schroeder et al.^20^ have discussed, local selection would not explain why this retrovirus became widespread “in almost all modern breeds of Eurasia since the retroviral insertions are … not causally linked with improved wool production”.

The frequency of **enJS5F16** has been quite constant from the Iron Age to Modern Period (in average 46%) and decreased to 36% in the Kihnu sheep. Most other reference populations, both NST and improvement breeds, had higher frequencies of this provirus, as did the Late Bronze Age sheep in Estonia.

The fourth provirus—**enJSRV-8**—was the rarest in all studied populations, often completely absent in modern breeds. Interestingly, no ancient Estonian sheep displayed this provirus integration, while in the Kihnu sheep it was present in almost a third of the tested animals (30%), a frequency similar to Texel (35%) and Leicester (28%) breeds. Therefore, the presence of this provirus in Kihnu populations could result from the nineteenth/twentieth century improvement of the local indigenous animals with these particular breeds or perhaps from the effects of recent inbreeding or genetic drift. Other breeds in the comparative dataset having this provirus were the Faroe (20%), Gute (18%), and Finnsheep (6%). For Gute sheep, for example, it has also previously been suggested that it is not a fully primitive breed but crossed with modern breeds, especially with Texel^16, 23^.

Overall, we identified a significant difference in the frequency of the four proviruses between modern Kihnu sheep and all ancient sheep (*χ*^2^ = 18.7, df = 3, *p* = 0.0003). Subsequent analyses of presence/absence of each proviruses independently indicates that this difference is attributable to significant increases in the frequencies of enJSRV-18 (*χ*^2^ = 7.00, df = 1, *p* = 0.008) and enJSRV-8 (*χ*^2^ = 19.4, df = 1, *p* = 0.00001) in modern Kihnu sheep; no significant difference were observed in the presence/absence of enJSRV-7 (*χ*^2^ = 1.13, df = 1, *p* = 0.287) or enJS5F16 (*χ*^2^ = 1.58, df = 1, *p* = 0.209) through time.

### Retrotype distribution

Chessa et al.^13^ described 15 retrotypes (R0–R14), of which 13 were found in Estonian sheep. Temporal view on the assigned retrotypes showed that eight retrotypes (R0–R7) were present in both ancient and modern populations in Estonia, while the remaining five (R8–R9, R11–R12, R14) were present only in Kihnu native sheep (Fig. 1; Supplementary Tables S1-5, S1-6 online). Moreover, the chi-squared test confirmed the retrotype distribution to be significantly different between the modern Kihnu and total ancient sample (*χ*^2^ = 39.24, df = 12, *p* = 0.0001).

**R0** and **R1**—with no provirus or with only enJSRV-7, respectively—have been described as the most primitive retrotypes^13^. R0 was present in almost all analysed NST breeds and in six individuals of the Kihnu sheep. R1 was much less frequent. The proportionally higher frequency of R1 in the most primitive breed Soay and in the Estonian Iron Age sheep seems to suggest loss of this retrotype in the course of time for several NST breeds. Importantly, R1 has been preserved in the Kihnu native sheep, together with only three other breeds: Rya, Icelandic, and the previously mentioned Soay. Neither of the two retrotypes occur in the analysed improvement breeds.

The most common retrotype—**R2**—is defined by the presence of enJSRV-18 only and is associated with the secondary introduction of improved wool sheep from south-west Asia^13^. It appeared to be absent in the most primitive breeds like Soay and Orkney and also in the Late Bronze Age individuals in Estonia. Since the Iron Age, the frequency of R2 increased and became most prevalent in Kihnu native sheep. Although R2 is associated with the secondary introduction, the Kihnu sheep of this retrotype present typical indigenous characteristics. It is interesting to note that among the 29 Leicester individuals tested by Chessa et al.^13^, none carried this retrotype.

**R3**, having only the insertion of enJS5F16, has been referred to as ‘Nordic’ retrotype and is widely present in NST breeds. Our results showed that also Estonian sheep have retained this retrotype since the Late Bronze Age. Although its frequency has decreased significantly in Kihnu native sheep, we still detected it in two individuals.

**R4**—a combination of enJSRV-18 and enJSRV-7—has been recorded in a high proportion around the Mediterranean basin, indicating a past selection for wool that occurred first in south-west Asia^13^. This retrotype was moderately present also in the NST breeds and in the tested Estonian sheep. Among the analysed improvement breeds, R4 was only present in Leicester in a very small proportion.

**R5** is a combination of the oldest provirus enJSRV-7 and the enJS5F16 (which on its own made the ‘Nordic’ retrotype R3) and can thus be regarded as more of a primitive or northern retrotype as well. This assessment was supported by its relatively high proportion among the Late Bronze Age and Iron Age individuals, as well as in Soay and Hebridean sheep, where this retrotype was predominant.

Although **R6** only appeared in our sample set during the Iron Age, we cannot conclude that this retrotype was introduced to Estonia during that period. More likely, it was already present during the Late Bronze Age, but not sampled. The frequency of R6 increased in the Middle Ages and Modern Period, but decreased again in the Kihnu population. R6 was also present in most of the other NST breeds, including the most primitive Soay (although in only one tested individual), and also in improvement breeds.

During the Modern Period, a new retrotype—**R7**—appears in the Estonian sheep population, identified in one individual (142OaKar6) from the Karksi Castle in southern Estonia. Radiocarbon dating of this sample places it between 1682–1937 cal CE (95% confidence interval) (Supplementary Table S1-1, Text S2.1.5 online). Although wide-scale fine-wool breed improvement in Estonia started only in the first half of the nineteenth century^24^, we cannot rule out the import of new breeds in earlier periods. Large-scale breeding of fine-wool sheep in western Europe, especially in England and Spain, had started already from around the twelfth/thirteenth century and continued to influence the wool market in Europe until the eighteenth century^25, 26^. In Estonia, very first attempts have been recorded from the mid-seventeenth century ^24^. Based on the radiocarbon calibration curve there is only a 25.0% probability, however, that Karksi specimen dates from 1682–1737 cal CE; instead, there is a 69.5% probability, that it dates from 1802–1937 cal CE, coinciding with the wider breed improvement that started in the nineteenth century and continued into the first half of the twentieth century. The relation of R7 to improved breeds is also reflected by the fact that R7 seemed to be less frequent in the NST breeds, but more frequent in Texel and Leicester. Therefore, we can conclude that based on the material analysed in this study, R7 is likely a modern introduction, related to recent breed improvement.

Five retrotypes—**R8–R9, R11–R12, R14**—are all determined by inclusion of enJSRV-8, which, as described above, was totally absent in ancient sheep and in most of the NST breeds, while abundant in British breeds and also in Kihnu sheep. Therefore, the presence of these retrotypes in Kihnu sheep could refer to some degree of gene flow from British breeds (Leicester and Texel in the current dataset) within the last two hundred years. Among NST breeds, few individuals of Finnsheep, Gute, and Faroe sheep also carried these retrotypes.

## Discussion

### The origins and formation of the Northern Short-tailed sheep

The present study investigated retrovirus integrations in Estonian ancient and modern sheep in order to elaborate on the ancient origin of the Kihnu native sheep breed. We worked on the assumption that “populations sharing the same provirus in the same genomic location are de facto phylogenetically related”^13^. Comparing the Kihnu native sheep to ancient samples and reference breeds of the NST, we confirmed some existing assumptions regarding the development of sheep populations in northern Europe and most importantly, provided new evidence for phylogenetic relatedness and genetic continuity.

There have been several hypotheses regarding the origin, spread, and development of the NST sheep. Previous studies have suggested that in northern Europe there was an initial founder population that fragmented into isolated populations^5^ and that the NST sheep have survived since the Bronze Age (like the Soay) to the Middle Ages^25^. Over the course of time, these peripheral northern populations became distinct from modern breeds, and the same time, also lost their in-breed diversity^5, 27^. This loss of diversity seems to be reflected in our results of the provirus integrations as well. For example, having the main result of our study as a presumption—Estonian ancient and modern Kihnu native sheep share the same ancestry with the NST—we can assume that the sheep in the past would show the presence of primitive lineages with a higher frequency than the NST breeds who have survived until today. Indeed, our analysis of enJSRVs and their combined retrotypes in ancient populations—especially in the Late Bronze Age and Iron Age—indicated often a wider diversity of retrotypes in the past than we see in the native NST breeds. For example, several breeds in Finland and Sweden are missing some retrotypes we see in the ancient populations in Estonia. We propose that aside the possibility of sampling bias, this reflects the true loss of diversity over time. On the other hand, the Soay who is regarded to be the most primitive, has still preserved seven out of eight retrotypes that we found in ancient Estonian populations, with the exception of R2, which might have introduced to the other northern populations with the second migration wave.

The analysis of retroviruses in ancient populations gave us a unique possibility to assess the genetic diversity of the primitive breeds from a new angle. In turn, the outcome of this study confirmed that faunal remains, studied in zooarchaeology, are valuable not only in the studies of the past, but also of present, calling upon better management and sampling ethics for ancient faunal material^28^.

### Genetic history of Estonian sheep based on retroviral integrations

Our study revealed that there have been changes in the retrovirus diversity since the Late Bronze Age to present-day, with the frequency of enJSRV-18 increasing, enJSRV-7 decreasing, enJS5F16 remaining largely the same, and enJSRV-8 appearing only in recent times. Statistical analysis suggested that the presence/absence of enJSRV-7 and enJS5F16 between the Kihnu native sheep and the total ancient population in Estonia were similar, while the presence/absence of enJSRV-18 and enJSRV-8 were significantly different. These fluctuations seem to reflect the overall development of local sheep populations, influenced by historical factors like trade and the eighteenth/nineteenth century shift to modern breed improvement. The latter is clearly visible in the appearance of enJSRV-8 which was absent in archaeological individuals, but present in modern samples of the Kihnu native sheep breed.

Based on the four tested proviruses, we assigned retrotypes to each tested individual of Estonian ancient and modern sheep. We detected eight retrotypes (R0–R7) both in archaeological and modern Kihnu samples and called them therefore ‘ancient’. The remaining retrotypes (R8–R9, R11–R12, and R14) were present only in Kihnu samples and we called them therefore ‘novel’. Altogether, Kihnu native sheep had a very high diversity of retrotypes—altogether 13 out of the 15 described by Chessa et al.^13^, while none of the other analysed populations had this kind of diversity. Perhaps it can be attributed to the highest number of samples tested (n = 80), but it could also reflect the two-fold ancestry of the Kihnu native sheep: the presence of the oldest proviruses and primitive retrotypes that originate from ancient ancestry, together with novel retrotypes resulting from introgression with improved breeds. Based on the retroviral introgressions analysed in this study, it seems that this kind of crossing has also sporadically occurred in some other NST breeds. Unfortunately, it is impossible to say whether this introgression first happened in the distant past (e.g., affinities to the improved wool-sheep of the second wave, decrease/increase of certain proviruses due to animal husbandry practices and/or trade) or more recently (e.g., result of breed improvement, most probably over the last few hundred years). Importantly, however, crossings with modern breeds seem not to have been too extensive, because several lineages carrying the oldest proviruses and ancient retrotypes have been preserved in the Kihnu native sheep breed.

### Application of the study in ongoing breeding program

In light of our molecular results, Kihnu native sheep truly seem to be the descendants of the past populations in Estonia. Together with the previous works on microsatellites and mitochondrial DNA^2, 3, 5–7^, results from provirus analysis offered compelling evidence that the Kihnu native sheep have a shared ancestry with the local indigenous populations and with the NST breeds. Therefore, we argue that the Kihnu native sheep breed can be regarded as a member of the NST group.

Kihnu sheep have retained the most primitive retrotypes R0 and R1, which reflect their development in the peripheries of north-eastern Europe. Northern ancestry is further confirmed in the presence of the ‘Nordic’ retrotype R3 and also R5. However, not only the sheep of the first migration wave with primitive retrotypes are important. Sheep of the later migratory episode (‘wool sheep’) are also ancient and were a foundation for the great majority of present-day breeds. The ancestry of R2, R4, and R6 is clearly based in the past, in the Late Bronze Age, Iron Age, and Middle Ages, and these lineages are thus worth preserving as well.

Selection strategy by the Kihnu Native Sheep Society focuses on previously established genetic lineages and on the presence of indigenous traits, especially regarding horns and face. The results of the studied retroviral integrations have provided an additional aid to the selection strategy of the sheep society. The individuals carrying the seven ‘ancient’ retrotypes (R0–R6) are now used and selected for in the breeding program (see Supplementary Text S2.2.2 online), especially those carrying R0, R1, and R3. Attention is also given to morphological traits that comply with the retrotypes. Interestingly, desired indigenous phenotypic traits seem to be in accordance with the presence of primitive retrotypes. Sheep with morphological features characteristic of the Kihnu breed and now also with suitable retrotypes get more points in the breeding program and are more valued breeding animals. Among other selection criteria, the Kihnu Native Sheep Society is now focusing its attention on the use of individuals with primitive retrotypes by trying to produce more offspring from these individuals and by combining new lineages. Therefore, thanks to this study, the sheep breeders are directly incorporating molecular information into conservation decisions.

## Supplementary information

Supplementary Tables.

Supplementary Information.

## Data Availability

The main data generated or analysed during this study are included in this published article and its supplementary information files. Additional details of the datasets are available from the corresponding author on reasonable request.
