# Supplementary material for: Retroviral analysis reveals the ancient origin of Kihnu native sheep in Estonia: implications for breed conservation
Source: Sci Rep. 2020 Oct 15;10:17340. doi: 10.1038/s41598-020-74415-z (PMC7566594; doi:10.1038/s41598-020-74415-z)
Supplement: Supplementary file 2 — Supplementary Information. [file 41598_2020_74415_MOESM2_ESM.pdf]

Retroviral analysis reveals the ancient origin of Kihnu native sheep in Estonia: implications for breed conservation

Eve Rannamäe, Urmas Saarma, Anneli Ärmpalu-Idvand, Matthew D. Teasdale, Camilla Speller

Supplementary information S2 Text

S2.1. Lab work

S2.1.1. Estonian samples

Altogether 80 modern and 83 ancient samples from Estonia were selected for this study. Ancient samples originate from various archaeological sites from the Late Bronze Age to Modern Period, modern samples are all from the Kihnu native sheep breed (Figure S2-1).

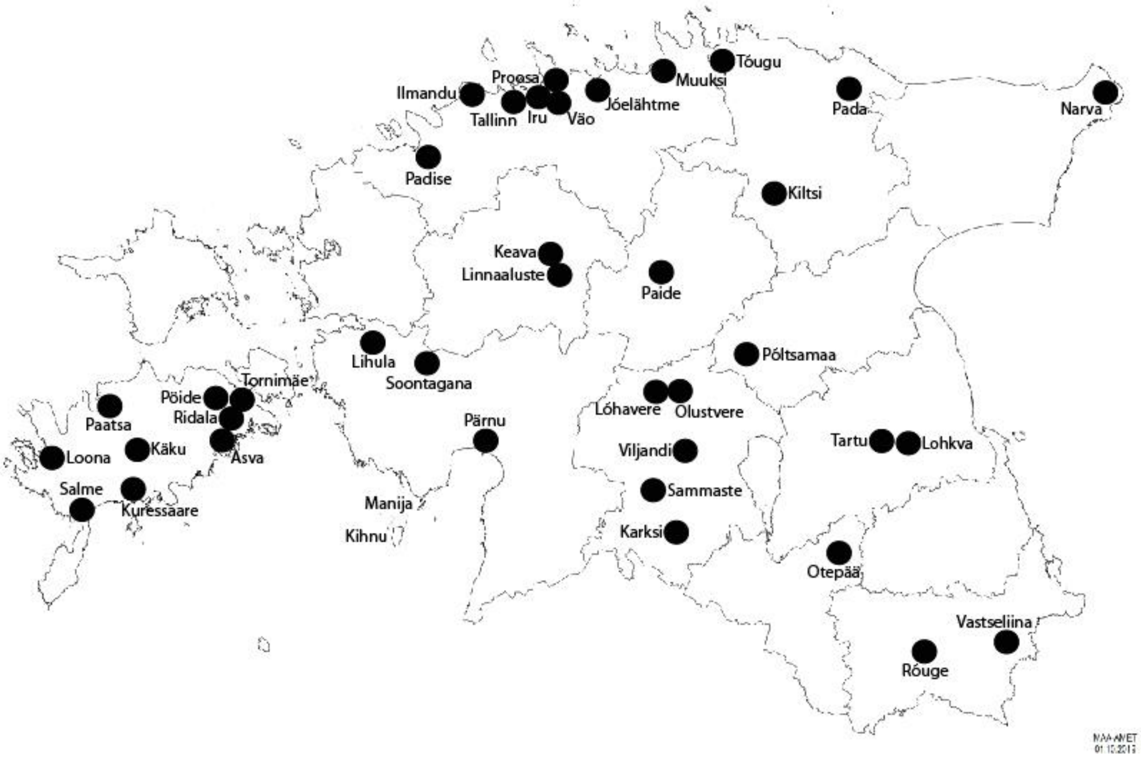

Figure S2-1. Archaeological sites of ancient sheep samples (n=83) analysed in this study. The samples of Kihnu sheep (n=80) were collected from the primary population and from the collection herds in Kihnu and Manija islands and in mainland south-west Estonia (Pärnu County). The Kihnu native sheep breed is named after the Kihnu Island. Map by Geoportal [22]. Image processing: Adobe Illustrator CS5 v.15 [23].

S2.1.2. Sampling and extraction protocol for modern DNA

The blood samples were collected to 9 mL vacutainers with EDTA (Greiner BioOne, Kremsmünster, Austria) and stored at -20°C until DNA extraction. DNA was extracted from 200 µl of blood using the High Pure PCR Template Preparation Kit (Roche Diagnostics), following the manufacturer's protocol. DNA extraction was conducted at the Department of Zoology, University of Tartu, Estonia. PCR and post-PCR work was conducted at BioArCh, University of York, United Kingdom.

S2.1.3. Sampling and extraction protocol for ancient DNA

All pre-PCR work was conducted in the ancient DNA (aDNA) laboratory, separate from the PCR and post-PCR laboratories at BioArCh, University of York, United Kingdom. Sample preparation and DNA extraction followed the silica spin-column protocol [1] with slight modifications. Non-disposable equipment was decontaminated between the samples, and latex gloves and protective clothing were worn when handling the specimens.

Thirteen sheep bones were subsampled using a sterile saw blade. Subsampled bones were soaked in 6% sodium hypochlorite for 5 min, rinsed in HPLC water three times, and UV irradiated for 20 min on two sides. The bone piece was ground in a mortar using a pestle, and 100–150 mg of bone powder was weighed out for the following extraction.

To demineralize the sample, 1 ml of lysis buffer with a concentration of 0.5M EDTA and 0.5 mg/ml Proteinase K was added and pre-digested at 37°C for 45 min. After that, the supernatant was removed and another 1.8 ml of the lysis buffer was added to the sample and digested at 50°C overnight. After incubation was complete, the sample was centrifuged at 13000 rpm for 5–10 min until the bone powder was separated from the buffer solution. To concentrate the sample, the supernatant was transferred to an Amicon™ Ultra Centrifugal Filter 10K MWCO (Merck, Darmstadt, Germany) and centrifuged at 4400 rpm for at least 90 min, until the preferred concentrated volume left in the membrane was 50–100 µl. QIAGEN QiaQuick MinElute kits were used for DNA purification. To bind the DNA, around 500–650 µl (5X the sample volume) of PB buffer was added to the sample, transferred to a MinElute column, and centrifuged for 1 min at 6500 rpm. The flow-through was discarded. To wash the DNA, 500 µl of PE buffer was added to the column, centrifuged for 1 min at 6500 rpm, and flow-through discarded. Another 500 µl of PE buffer was added to the column, centrifuged for 1 min at 13000 rpm, and flow-through discarded. To dry the column, it was centrifuged for 1 min at 13000 rpm and then transferred to a collection tube. To elute the DNA, 30 µl of heated (65°C) EB buffer was loaded to the column, incubated at room temperature for 5–10 min, and centrifuged for 1 min at 13000rpm. For the second elution, another 80 µl of EB was loaded to the column, incubated, and centrifuged. DNA was stored in safe-lock tubes at -20°C. For this study, the second elution of DNA was used for PCR amplifications.

S2.1.4. Zooarchaeology by mass spectrometry (ZooMS)

For five samples – 158-OaTal4, 162-OaEka4, 167-OaAsva4, 169-OaRid4, 170-OaRid5 – species ID had to be confirmed due to high similarities between sheep and goat morphology. This was done with collagen fingerprinting through mass spectrometry or zooarchaeology by mass spectrometry (ZooMS), which is a minimally destructive method for species identification.

Work was conducted at BioArCh, Department of Archaeology, University of York, United Kingdom, following a conventional destructive ZooMS method [2]. Briefly, the specimens were sampled for 15–30 mg of small bone pieces, demineralized in 0.6M hydrochloric acid, and washed once in 200 µl 0.1M sodium hydroxide and trice in 200 µl 50 mM ammonium bicarbonate solution pH 8.0. After one-hour incubation in 100 µl ammonium bicarbonate at 65°C, 50 µl of a sample was digested with 1 µl trypsin by incubating overnight at 37°C. Following incubation, 1 µl of 5% trifluoroacetic acid solution (TFA) was added to terminate trypsin activity. Peptides were extracted using a C18 ZipTip pipette tip (Millipore) treated with 0.1% TFA washing solution and 50% acetonitrile / 0.1% TFA conditioning solution, and then eluted with 50 µl conditioning solution. One microliter of the sample was spotted on a ground steel plate in triplicate, mixed with 1 µl α-cyano-4-hydroxycinnamic acid matrix solution. The plate was run on a calibrated Bruker Ultraflex III MALDI TOF/TOF mass spectrometer. Three spectra of each sample were averaged and analysed in mMass [3, 4, 5]. Individual peptides were identified manually according to previously published markers [6, 7, 8].

S2.1.5. Radiocarbon dating

Most samples were dated based on archaeological context, that is, through associated finds and site stratigraphy. Five samples with unclear context were radiocarbon dated by AMS in SUERC Radiocarbon Dating Laboratory and one sample in the Leibniz Laboratory for Radiometric Dating and Stable Isotope Research, Christian-Albrechts-University of Kiel – all calibrations according to IntCal20 atmospheric curve [9]; OxCal v.4.4.2 [10]; r:5. All six samples gave a successful and expected result (Supplementary Table S1-1 online). The radiocarbon date for sample 142OaKar6 is discussed in more detail in the main text (see Results – Retrotype distribution), and therefore, we present the calibration curve here (Figure S2-2). For 14 samples, dating results have previously been reported in Rannamäe et al. [11, 12], but recalibrated here according to IntCal20 atmospheric curve [9]; OxCal v.4.4.2 [10]; r:5.

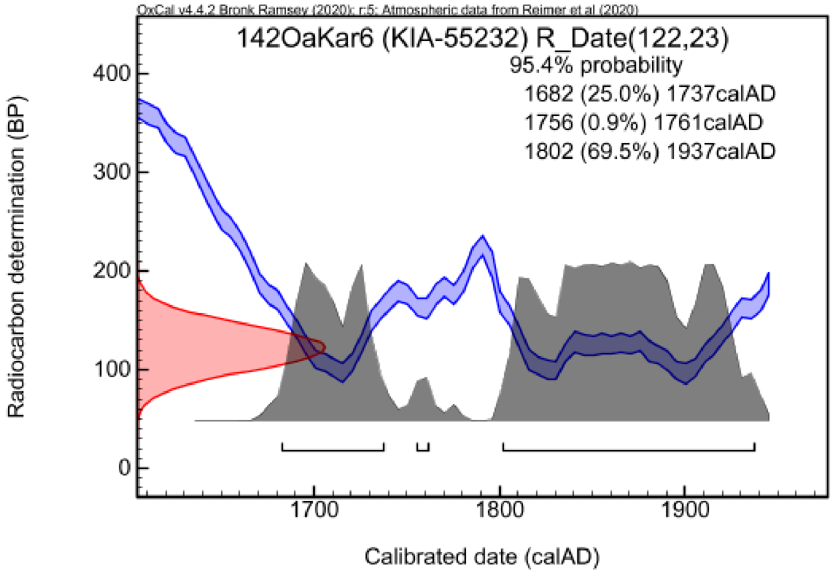

Figure S2-2. Calibration curve and age range for the sample 142OaKar6 from Karksi Castle. The Y-axis indicates the radiocarbon determination (distribution in red), while the solid grey distribution indicates the likelihood of different possible calendar date age ranges. The calibration curve was produced using OxCal v.4.4.2 [10].

S2.1.6. Primers and conditions for polymerase chain reaction (PCR)

We used PCR primers designed to amplify short degraded DNA fragments. Some primers had been previously published [13] and some were designed specifically for this study (Table S2-1). The same primers were used for both ancient and modern DNA. With a set of three primer pairs, three regions of each provirus were targeted: the 5′ and 3′ long terminal repeats (LTRs) including the genomic flanking region of the host; and the empty locus (EL), which is the empty genomic insertion site, that is, only the genomic flanking regions of the host (for schematic representation of the PCRs, see [14, 15]). Essentially, only the presence of one of the two LTR regions (5′LTR or 3′LTR) would be enough to confirm the provirus insertion in the host genome: if 5′LTR is present/absent, then 3′LTR has to be the same and vice versa. Based on the test PCRs on modern samples, we decided to focus on amplifying the 5′LTR region and the EL, because the 3′LTR seemed to give partially unclear results (smear in gel images). However, if it was necessary to authenticate the presence/absence of the 5′LTR, we also tested the 3′LTR.

Table S2-1. List of primers used in this study.

Locus	Provirus name	Primer pair	Primer sequence	Frag. length	Reference sequence	Tm (salt adjusted)	Reference
5′LTR	enJSRV-18	V-18_Flank5F	TTCGTTTCTTAGGCGCTCTGA	80 bp	EF680301.1 [14]	59.5°C	modified after [13]
		LTR_5R	AGCTCCCAAGACTTAACCCT			58.4°C	[13]
	enJSRV-7	V-7_Flank5F	GCTTGAACGTCAAGGCAGTG	80 bp	EF680298.1 [14]	60.5°C	This study
		LTR_5R	AGCTCCCAAGACTTAACCCT			58.4°C	[13]
	enJSRV-5F16	V-5f16_Flank5F	CGTGTTGAACTCTTTGGCAGT	80 bp	AF136224.1 [24]	59.5°C	This study
		LTR_5R	AGCTCCCAAGACTTAACCCT			58.4°C	[13]
	enJSRV-8	V-8_Flank5F	TGAGGAAACTAGCAAAAAGAGGT	80 bp	EF680306.1 [14]	59.2°C	This study
		LTR_5R	AGCTCCCAAGACTTAACCCT			58.4°C	[13]
3′LTR	enJSRV-18	V-18 _Flank3R	TCGGACATAGCCACGGTGT	100 bp	EF680301.1 [14]	59.5°C	This study
		LTR_3F	TCTTGCTTGTGCTGGCCG			58.4°C	
	enJSRV-7	V-7_Flank3R	ACAGGAAAGGCGAGAGGAAC	100 bp	EF680298.1 [14]	60.5°C	This study
		LTR_3F	TCTTGCTTGTGCTGGCCG			58.4°C	
	enJSRV-5F16	V-5f16_Flank3R	CCAGGGTTGGAAAGATCCC	100 bp	AF136224.1 [24]	59.5°C	This study
		LTR_3F	TCTTGCTTGTGCTGGCCG			58.4°C	
	enJSRV-8	V-8_Flank3R	GGTAGCATAGTTCGGTGGTTAA	100 bp	EF680306.1 [14]	60.1°C	This study
		LTR_3F	TCTTGCTTGTGCTGGCCG			58.4°C	
empty locus	enJSRV-18	V-18_Flank5F	TTCGTTTCTTAGGCGCTCTGA	118 bp	EF680301.1 [14]	59.5°C	modified after [13]
		V-18_Flank3R	TCGGACATAGCCACGGTGT			59.5°C	This study
	enJSRV-7	V-7_Flank5F	GCTTGAACGTCAAGGCAGTG	118 bp	EF680298.1 [14]	60.5°C	This study
		V-7_Flank3R	ACAGGAAAGGCGAGAGGAAC			60.5°C	
	enJSRV-5F16	V-5f16_Flank5F	CGTGTTGAACTCTTTGGCAGT	118 bp	AF136224.1 [24]	59.5°C	This study
		V-5f16_Flank3R	CCAGGGTTGGAAAGATCCC			59.5°C	
	enJSRV-8	V-8_Flank5F	TGAGGAAACTAGCAAAAAGAGGT	118 bp	EF680306.1 [14]	59.2°C	This study
		V-8_Flank3R	GGTAGCATAGTTCGGTGGTTAA			60.1°C	

PCR for modern samples. PCR amplification was carried out in 10 µl reaction mix containing: PCR-grade water, 1X QIAGEN Multiplex PCR Kit (Qiagen), 0.2 µM of each primer, and 1.0 µl of DNA template. PCR was conducted as follows: 15 min denaturing at 95°C, 35 three-step cycles of 94°C for 30 sec, 60°C for 90 sec, and 72°C for 30 sec; followed by a final extension of 72°C for 10 min.

PCR for ancient samples. PCR amplification was carried out in 30 µl reaction mix which contained: 1.5X PCR buffer, 2.5 mM MgCl2, 0.2 mM dNTP, 1 mg/ml BSA, 0.3 µM of each primer, 2.5 U AmpliTaq Gold DNA Polymerase LD (Applied Biosystems), and 3.0 µl of DNA

template. PCR was conducted as follows: 10 min denaturing at 95°C, 60 three-step cycles of 95°C for 30 sec, 62°C for 30 sec, and 72°C for 1 min; followed by a final extension of 72°C for 7 min.

Electrophoresis. Amplified products were detected by electrophoresis on 2.4% agarose gel containing 0.5X TBE buffer and SYBR Safe DNA Gel Stain (Invitrogen). Electrophoresis was for 30 min at 100V/400 mA. The samples were loaded with 50 bp DNA Ladder (Invitrogen) and the bands were viewed under transilluminator.

S2.1.7. Authentication

Contamination of PCR was monitored by the use of negative controls for every primer pair in each PCR. Preparation of PCR reactions was done separately from the post-PCR work.

To test the quality of the primers, we amplified 5′LTR and EL of the 'ancient' enJSRV-6 in eight samples. enJSRV-6 is an 'ancient' enJSRV and thus fixed in all sheep [14, 16]. For this test, we used both previously published primers [15] and those from this study. All results were positive as expected. The primers designed for this study, suitable to amplify shorter fragments of the aDNA, gave even clearer gel images than the previously published primers and therefore we decided to use these primers for the rest of the modern DNA samples as well. To further verify the performance of the 5′LTR primers, we chose seven samples to repeat the PCR with the 3′LTR region. Consequently, all results agreed. Five PCR products were sequenced (Table S2-2), confirming the correct target region. For all the rest of the modern samples, one PCR was agreed to be sufficient for a positive amplification, while all negative amplifications were confirmed with a second PCR to monitor for allelic dropout as a cause of false negatives.

In ancient samples, we mostly focused on 5′LTR instead of the 3′LTR, because the latter gave occasionally poor results, especially for enJSRV-7 and enJS5F16. Poor function of the 3′LTR primers could derive from factors like duplication of the genomic DNA at the site of provirus integration or identical LTR regions within and between proviruses [14], but also of poor preservation of the DNA and difficulties in targeting ancient nuclear DNA with designated primers. For enJSRV-18 and enJSRV-8, the 3′LTR primers worked well, authenticating the results gained from 5′LTR. All the rest of the 5′LTR amplifications were repeated at least twice. Furthermore, 25 PCR products were sequenced to confirm some of the amplifications (Table S2-2). Only samples with high-confident results were selected for the analysis.

It is important to note that while the results from modern DNA samples can be considered reliable for the presence/absence of each provirus (supported by the quality of the DNA and several steps of authentication), results may be less reliable with ancient samples. In aDNA samples, it is almost impossible to confirm whether the amplification was negative because of the absent insertion or due to DNA degradation – even when PCRs were repeated several times. Nevertheless, we consider our results for the ancient samples reach a reliable level of confidence, not just because of the authentication steps taken, but also because of the distribution of the enJSRVs and retrotypes are consistent with previously published data.

Table S2-2. Sequencing results of the correct target regions of the enJSRVs. Primer sequences are included (marked in bold) and differences with reference sequence highlighted (in grey). For references, see Table S2-1.

Provirus	Position	Reference / Sample ID	Sequence
**enJSRV-18**	5′LTR	EF680301.1<br>12-OaOrdu2	**TTCGTTTCTTAGGCGCTCTGA**GCCTGATTGCATTTAGGAAGTGCGGGGGACGACCCGTGA**AGGGTTAAGTCTTGGGAGCT**<br>**TTCGTTTCTTAGGCGCTCTGA**GCCTGANTGCANTTAGGAAGTGCGGGGNANGACCCGTGA**AGGGTTAAGTCTTGGGAGCT**
	3′LTR	EF680301.1<br>12-OaOrdu2	**TCTTGCTTGTGCTGGCCG**CGGCAGGAAGAGCTGTGGGCTGGCTCTGGGCTCTGGCACTTGGGCACAGGGACCCTCTTCTGG**ACACCGTGGCTATGTCCGA**<br>**TCTTGCTTGTGCTGGCCG**CGGCAGGAAGAGCTGTGGGCTGGCTCT-GGCTCTGGCACTTGGGCACAGGGACCCTCTTCTGG**ACACCGTGGCTATGTCCGA**
	EL	EF680301.1<br>12-OaOrdu2	**TTCGTTTCTTAGGCGCTCTGA**GCCTGATTGCATTTAGGAAGGGAAGAGCTGTGGGCTGGCTCTGGGCTCTGGCACTTGGGCACAGGGACCCTCTTCTGG**ACACCGTGGCTATGTCCGA**<br>**TTCGTTTCTTAGGCGCTCTGA**GCCTGATTGCATTTANNNNNGGAAGAGCTGTGGGCTGGCTCTGGGCTCTGGCACTTGGGCACAGGGACCCTCTTCTGG**ACACCGTGGCTATGTCCGA**
**enJSRV-7**	5′LTR	EF680298.1<br>118-OaMusu2<br>ML2	**GCTTGAACGTCAAGGCAGTG**CAGGGAAAACAGGTTCCTCTGTGCGGGGGACGACCCGTAA**AGGGTTAAGTCTTGGGAGCT**<br>**GCTTGAACGTCAAGGCAGTG**CAGGNAAAACAGGT-CCTCTGTGCGGGGNANGACCCGTAA**AGGGTTAAGTCTTGGGAGCT**<br>**GCTTGAACGTCAAGGCAGTG**CAGGGAAAACAGNTNCCTCTGTGCGGGGGANGACCCGTAA**AGGGTTAAGTCTTGGGAGCT**
	3′LTR	EF680298.1<br>118-OaMusu2<br>ML2	**TCTTGCTTGTGCTGGCCG**CGGCACCTCTGGAATGGACTGACACATCTGGGGGACTCTTGGAATGGTGGCACGACCCTGGA**GTTCCTCTCGCCTTTCCTGT**<br>**TCTTGCTTGTGCTGGCCG**CGGCACCTCTGGAATGGACTGACACATCTGGG<br>**TCTTGCTTGTGCTGGCCG**CGGCACCTCTGGAANGGACTGACNNNTCTGGGGNANTCTTGGANTGGTGGCACGACCCTGGA**GTTCCTCTCGCCTTTCCTGT**
	EL	EF680298.1<br>118-OaMusu2<br>ML2	**GCTTGAACGTCAAGGCAGTG**CAGGGAAAACAGGTTCCTCTGCCTCTGGAATGGACTGACACATCTGGGGGACTCTTGGAATGGTGGCACGACCCTGGA**GTTCCTCTCGCCTTTCCTGT**<br>**GCTTGAACGTCAAGGCAGTG**CAGGGAAAACAGGNNNNNNTNCCTCTGGAATGGACTGACACATCTGGGGGACTCTTGGNATGGTGGCACGACCCTGGA**GTTCCTCTCGCCTTTCCTGT**<br>**GCTTGAACGTCAAGGCAGTG**CAGGGAAAACAGGTTCCTCTG--T--GGAATGGACTGACACATCTGGGGGACTCTTGGAATGGTGGCACGACCCTGGA**GTTCCTCTCGCCTTTCCTGT**
**enJS5F16**	5′LTR	AF136224.1<br>118-OaMusu2<br>ML2	**CGTGTTGAACTCTTTGGCAGT**-CTGCCAGGCTCCTCTGTCCCTGCGGGGGACGACCCGTGA**AGGGTTAAGTCTTGGGAGCT**<br>**CGTGTTGAACTCTTTGGCAGT**TCTGCCAGNCTCCTCTGTCNCTGCGGGNNANGACCNGTGN**AGGGTTAAGTCTTGGGAGCT**<br>**CGTGTTGAACTCTTTGGCAGT**-CTGCCAGNCTCCTCTGTC--TGCGGG--ACGACCCGTGA**AGGGTTAAGTCTTGGGAGCT**
	3′LTR	AF136224.1<br>118-OaMusu2	**TCTTGCTTGTGCTGGCCG**CGGCACTGTCCATGGGATTTCCCAGGCAAGAATACTGGAGTGGGCTGCCATTTCCTTCTCCAC**GGGATCTTTCCAACCCTGG**<br>**TCTTGCTTGTGCTGGCCG**CGGCACTGTCCATGGGATT
	EL	AF136224.1<br>118-OaMusu2<br>ML2	**CGTGTTGAACTCTTTGGCAGT**CTGCCAGGCTCCTCTGTCCCCTGTCCATGGGATTTCCCAGGCAAGAATACTGGAGTGGGCTGCCATTTCCTTCTCCAC**GGGATCTTTCCAACCCTGG**<br>**CGTGTTGAACTCTTTGGCAGT**CTGCCAGGCTCCTCTGTCCNNNNNNNATGGGATTNCCCAGGCAAGAATACTGGAGTGGGCTGCCATTTCCTTCTCCAC**GGGATCTTTCCAACCCTGG**<br>**CGTGTTGAACTCTTTGGCAGT**CTGCCAGGCTCCTCTGTCCNNNNNNNATGGGATTTCCCAGGCAAGAATACTGGAGTGGGCTGCCATTTCCTTCTCCAC**GGGATCTTTCCAACCCTGG**
**enJSRV-8**	EL	EF680306.1<br>4-OaJaan1	**TGAGGAAACTAGCAAAAAGAGGT**TAGGTATTTAAGGTAAGGGTAAGGTTGTTCAGATAGAAAGGGGAAGAATAGTTTGGCTTTAGAATGTGTACTC**TTAACCACCGAACTATGCTACC**<br>**TGAGGAAACTAGCAAAAAGAGGT**TAGGTATTTAAGGTAAGGNNNNNGTTGTTCAGATAGAAAGGGGAAGAATAGTTTGGCTTTAGAATGTGTACTC**TTAACCACCGAACTATGCTACC**

S2.2. Results

S2.2.1. Zygosity

As the sheep is a diploid organism, the retrovirus insertion could be present in both chromosomes (homozygous) or in only one of them (heterozygous). In case of heterozygous individuals, we considered the insertion dominant (following [15, 17]). Presence/absence of the insertion was shown as 1 or 0, respectively (Table S2-3).

Table S2-3. The strategy of assessing the presence/absence of the retrovirus insertion based on the amplification result (positive/negative) of each of the three target regions.

Target region	PRESENT – 1<br><br>homozygous – insertion on both chromosomes, no empty locus	PRESENT – 1<br><br>heterozygous – insertion on one chromosome, empty locus on the other	ABSENT – 0<br><br>no insertions, only empty locus
5′LTR	pos.	pos.	neg.
3′LTR	pos.	pos.	neg.
EL	neg.	pos.	pos.

In both modern and ancient sheep, for enJSRV-18 and enJS5F16, both homozygous and heterozygous individuals were found, while for enJSRV-7, all individuals were heterozygous. For enJSRV-8, both homozygous and heterozygous individuals were present among Kihnu population, while in ancient samples this retroviral integration was not present in any time period (Tables S2-4, S2-5).

Table S2-4. Zygosity of the four analysed enJSRVs in Kihnu sheep (number of individuals and frequency).

Zygosity	Number of individuals				Frequency			
	enJSRV-7	enJSRV-18	enJS5F16	enJSRV-8	enJSRV-7	enJSRV-18	enJS5F16	enJSRV-8
1/1 (homozygous)	0	28	2	1	0,0%	35,0%	2,5%	1,3%
0/1 (heterozygous)	11	32	27	23	13,8%	40,0%	33,8%	28,8%
0/0 (no ERV)	69	20	51	56	86,3%	25,0%	63,8%	70,0%
Total:	80	80	80	80	100,0%	100,0%	100,0%	100,0%

Table S2-5. Zygosity of the four analysed enJSRVs in ancient sheep (number of individuals and frequency).

Zygosity	Number of individuals				Frequency			
	enJSRV-7	enJSRV-18	enJS5F16	enJSRV-8	enJSRV-7	enJSRV-18	enJS5F16	enJSRV-8
1/1 (homozygous)	0	3	5	0	0,0%	5,7%	9,4%	0,0%
0/1 (heterozygous)	11	25	20	0	20,8%	47,2%	37,7%	0,0%
0/0 (no ERV)	42	25	28	53	79,2%	47,2%	52,8%	100,0%
Total:	53	53	53	53	100,0%	100,0%	100,0%	100,0%

S2.2.2. Retrotype examples among the Kihnu native sheep

There were 18 maternal lineages in the primary population of the Kihnu native sheep. Currently (in August 2020), 12 of these lineages are still present (i.e., their living descendants) and altogether 480 breeding animals are being used in the breeding program.

Kihnu native sheep shared eight retrotypes with the ancient individuals from the Late Bronze Age to the Modern Period (R0–R7). Of these, R7 is related to the Modern Period and possibly to improvement breeds. The remaining seven retrotypes (R0–R6) we considered inherent to the indigenous population in Estonia and thus worth preserving in the current breeding program. Interestingly, individuals from these seven retrotypes feature good primitive morphological traits, which were already being valued and selected in the breeding program. Among the sheep tested for this study, some were from the primary population and some were their descendants (Supplementary Table S1-2 online). Importantly, many of those descendants belong to primitive or ancient retrotypes, indicating good selection strategy already in the earlier days of the breeding program. Knowing the retrotypes of the tested breeding animals and combining this information with preferred phenotypic traits allows even better selection in the future.

Kihnu native breed is characterized by small hardy body build, short tail, chance of horns in both males and females, occasional occurrence of wattles, double layered coat of hairy outercoat and woolly undercoat, primitive fleece structure, variety of colours, prolificacy and strong maternal instinct, adaptation to local environmental conditions, and resistance to diseases and parasites. In general, native sheep have developed during hundreds of years in mutual relationship with the local environment and their morphological traits are in conjunction with local adaptations. Moreover, indigenous animals have adapted to use vegetation most economically and today the native sheep are helping to preserve semi-natural environments and biodiversity. Porter et al. have stated that "the difficulty of realizing and developing these traits, without incurring the inherent dangers of commercial exploitation, is a problem that must be addressed in the 21st century" [18]. Therefore, it is very important that all these factors together with morphological traits and molecular information are considered in order to contribute to the conservation of overall diversity of the northern European sheep breeds.

The characteristics valued in the Kihnu native sheep breeding program are horns in both males and females, wattles, short tail, small size, slim legs, double layered coat, range of colours, and age-related changes in wool colour. Kihnu native sheep have six different colour types. The most typical is black with white markings, followed by white and so-called dalmatian, black, white with black markings, and so-called blackneck. The characteristics are regarded indigenous and come from a long-term adaptation to local climate and landscape. For example, the double layered coat is good for thermo- and hydroregulation, while slim legs allow fast and skilful movement on the landscape.

Short tail is the feature that in some of the previous studies and discussions has provoked questions regarding the position of the Estonian native sheep among other northern breeds. For the indigenous sheep in Estonia, the tail length has been described as variable or intermediate and probably a result of being crossed with long-tailed breeds [19, 20, 21]. So, how short should the tail be? Previous publications have stated different values for the allowed tail length and for the number of caudal vertebrae in NST sheep. Dýrmundsson & Niżnikowski wrote in 2010 that in the tail of short-tailed sheep there are usually 8–10 caudal vertebrae compared to 16–18 in long-tailed

sheep; however, in their statement they refer to rather old studies [21]. In a later publication of *Mason's Encyclopedia*, a tail length of 14 cm or less (as in the Soay) to 25 cm (as in the Hebridean) and with 12–14 caudal vertebrae are stated as suitable measures for the NST [18]. Interestingly, it is also noted that the number of caudal vertebrae in non-short-tailed sheep would be 20 or more [18], leaving a kind of 'grey' area in-between the number of 14 and 20. Moreover, the length of the tail is not considered as a precise indicator of the number of the caudal vertebrae [18]. According to the records of the Kihnu Native Sheep Society, live Kihnu sheep averagely possess a 21–22 cm long tail, which fits well into the limits described by Porter et al. [18]. But, on the other hand, our preliminary observations on the number of caudal vertebrae in Kihnu sheep show it to be in average between 14–16, and thus leading to another discrepancy in the morphology of the tail. Nevertheless, even though the tail in the Kihnu sheep is not as short as in several other NST breeds, it is very much shorter than in modern improvement breeds.

Below we give some examples of the tested Kihnu native sheep with their retrotype and phenotypic features. All photos are by A. Ärmpalu-Idvand, if not stated differently.

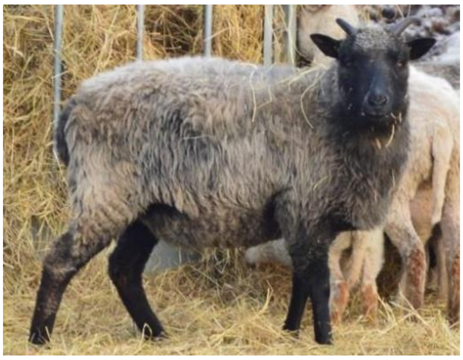

Retrotype 0. Vesuuvike (sample E8) – presence of sturdy horns in females.

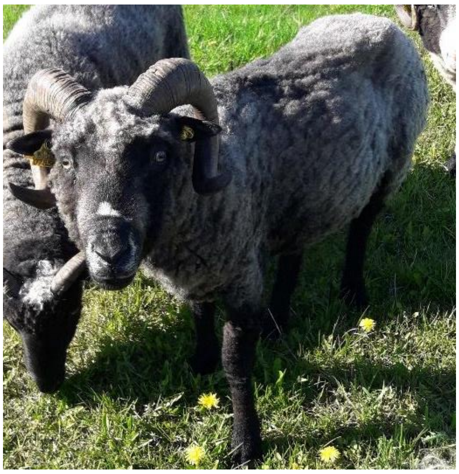

Retrotype 0. Variser (sample ML9) – large M-shaped horns in rams.

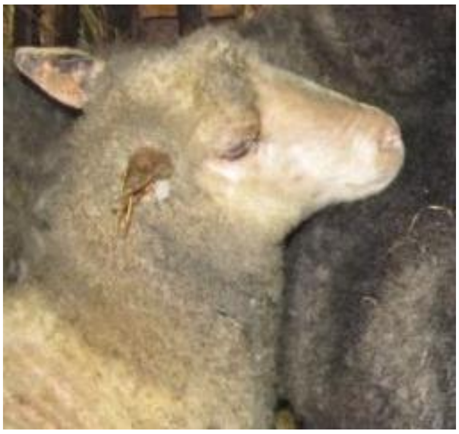

Retrotype 0. Viirpuu (sample E15) – white colour type with clean glossy face and glossy outercoat which seem to be indigenous characteristics.

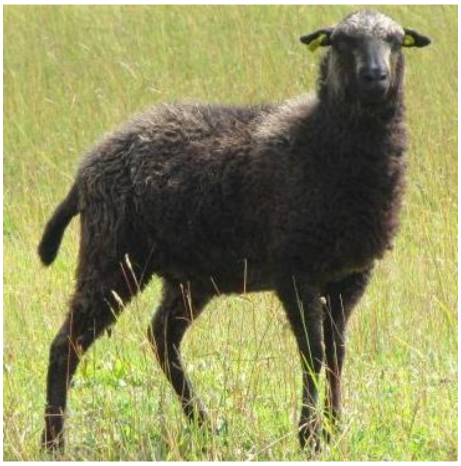

Retrotype 0. Krišnaiid (sample E4) – short tail and hairy outercoat.

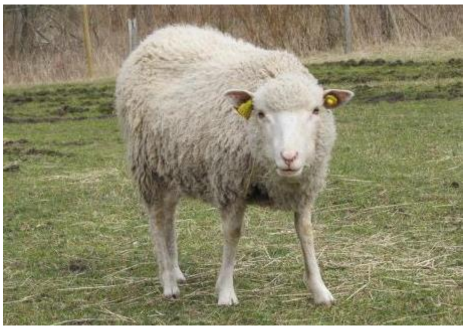

Retrotype 1. Luisi (sample ML21) – white colour, very short tail, and clean glossy face which seems to be an indigenous characteristic.

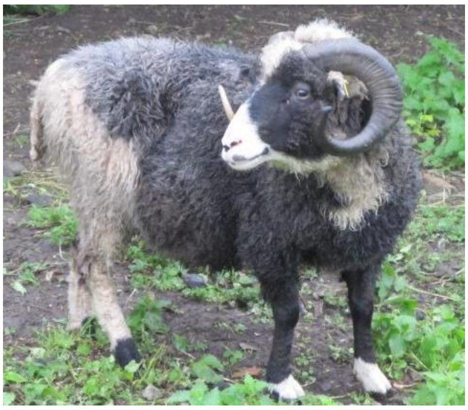

Retrotype 2. Lexus (sample ML10) – although not considered a primitive retrotype, R2 as an ancient retrotype, present in Estonia since the Late Bronze Age, still features good characteristics like large horns, clean face, short tail, glossy outercoat, clear double layered coat, and small size. Lexus here had all characteristic features except the wattles. Lexus was a descendant of Liina (sample E1, R4) and his own descendants are regarded very good.

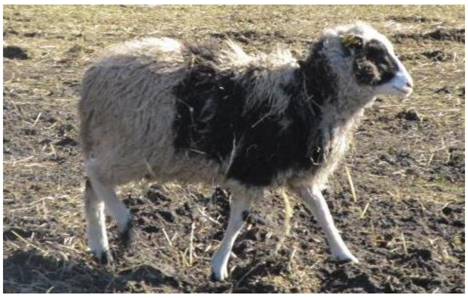

Retrotype 3. Tundra (sample ML5) – a dalmatian colour type, highly regarded in the breeding program. Dalmatians among the Kihnu native sheep seem to have a specific feature of very clear patches and with white patches that turn black-spotted with age. To our knowledge, these features are not characteristic to other Scandinavian sheep.

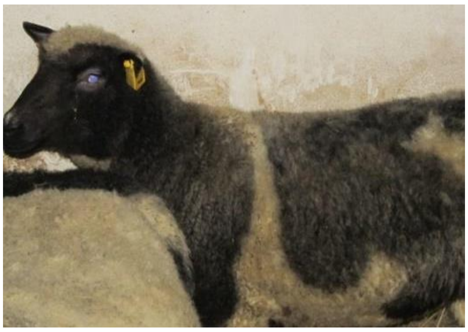

Retrotype 3. Võilill (sample E14) – a dalmatian colour type, highly regarded in the breeding program.

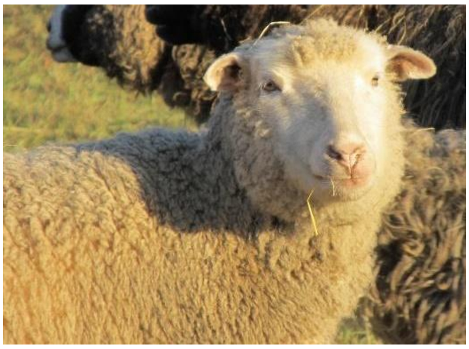

Retrotype 4. Luise (sample ML15) – white colour type with clean glossy face which seems to be an indigenous characteristic.

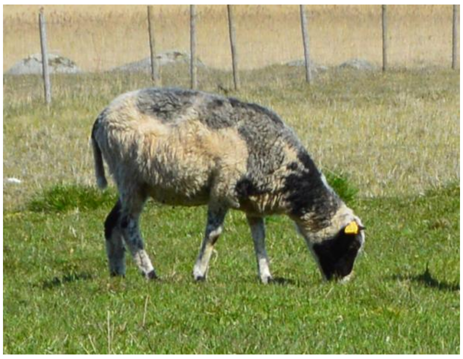

Retrotype 4. Lipsi (sample ML27) – short tail and a white with black markings colour type.

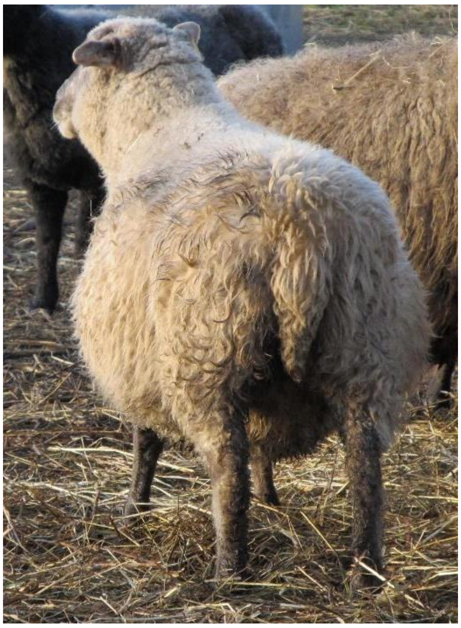

Retrotype 5. Kullerkupp (sample ML3) – short tail and glossy outercoat.

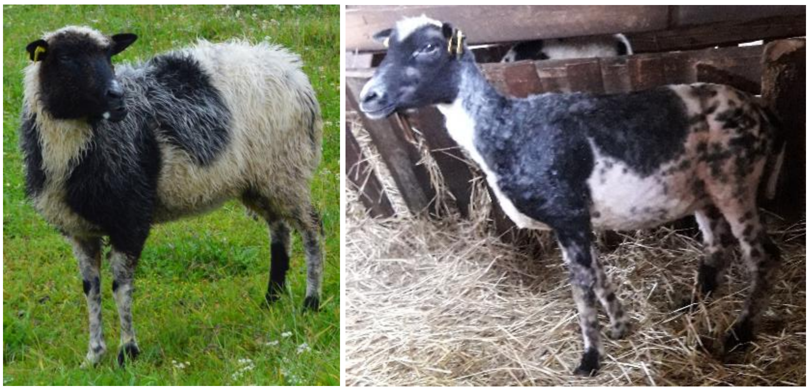

Retrotype 6. Viru (sample ML16) with and without fleece – a dalmatian colour type with characteristic black spots on the white patches.

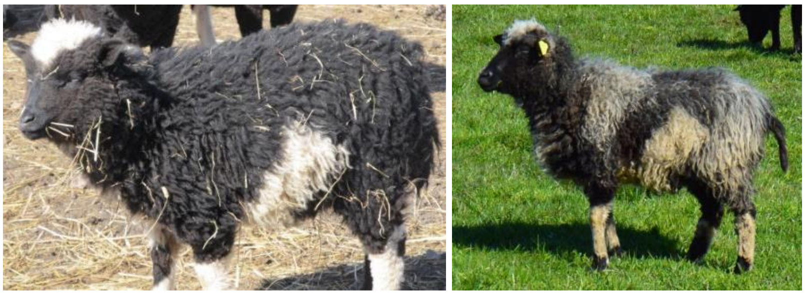

Retrotype 6. Virulaulik (sample ML17) as a juvenile and adult – very typical black with white markings colour type, which has changed with age.

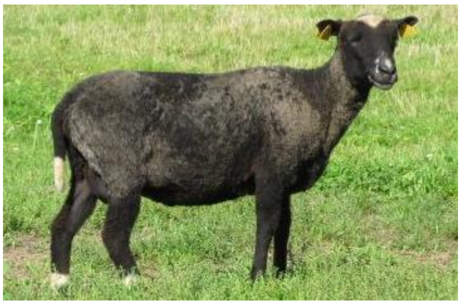

Retrotype 6. Musi (sample E19) – very typical black with white markings colour type.

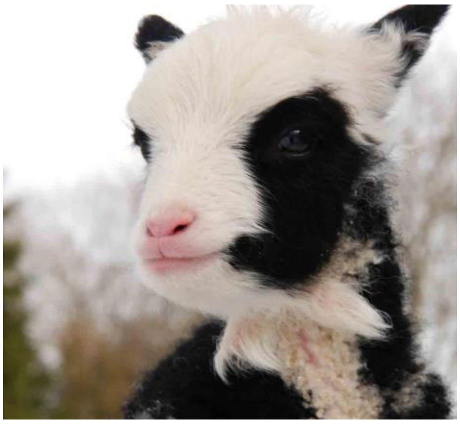

Retrotype 6. Urti (sample LA46) – very indigenous characteristics are wattles, clearly visible in juvenile age. Photo: S. Vahur.

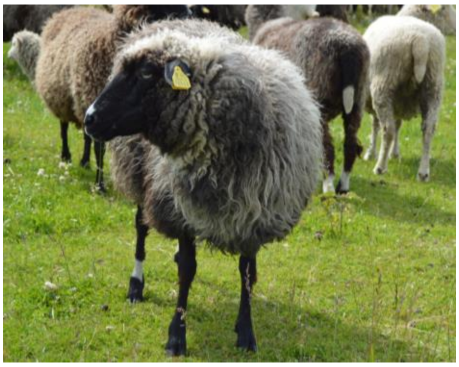

Retrotype 6. Kristall (sample E6) – slim legs.

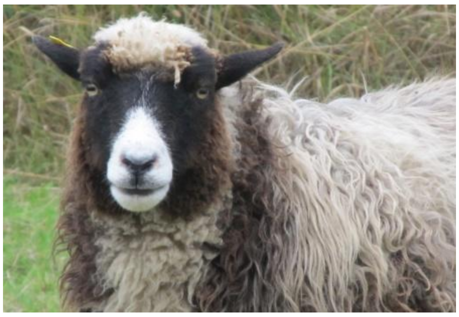

Retrotype 7. Kruusa (sample ML2) – very typical black with white markings colour type, which has changed with age.

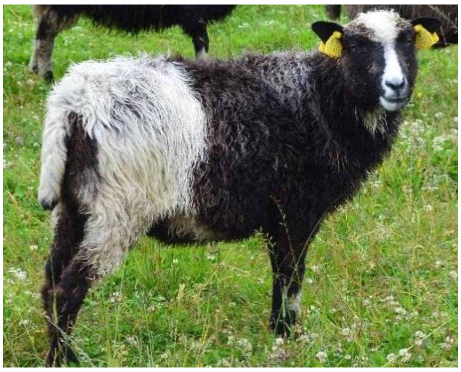

Vaskkael (not sampled for enJSRVs) – an example of blackneck color type.

References

1. Yang, D. Y., Eng, B., Waye, J. S., Dudar, J. C. & Saunders, S. R. Technical note: improved DNA extraction from ancient bones using silica-based spin columns. *Am. J. Phys. Anthropol.* **105(4)**, 539–543 (1998).
2. McGrath, K. et al. Identifying Archaeological Bone via Non-Destructive ZooMS and the Materiality of Symbolic Expression: Examples from Iroquoian Bone Points. Sci. Rep. **9**, 11027; 10.1038/s41598-019-47299-x (2019).
3. Strohalm, M., Hassman, M., Košata, B. & Kodíček, M. mMass Data Miner: an Open Source Alternative for Mass Spectrometric Data Analysis. *Rapid Commun. Mass. Spectrom*. **22(6)**, 905–908 (2008).
4. Strohalm, M., Kavan, D., Novák, P., Volný, M. & Havlíček, V. mMass 3: A Cross-Platform Software Environment for Precise Analysis of Mass Spectrometric Data. *Anal. Chem.* **82(11)**, 4648–4651 (2010).
5. Niedermeyer, T. H. J. & Strohalm, M. mMass as a Software Tool for the Annotation of Cyclic Peptide Tandem Mass Spectra. *PLoS ONE* **7(9)**, e44913; 10.1371/journal.pone.0044913 (2012).
6. Buckley, M., Collins, M., Thomas-Oates, J. & Wilson, J. Species identification by analysis of bone collagen using matrix-assisted laser desorption/ionisation time-of-flight mass spectrometry. *Rapid Commun. Mass. Spectrom*. **23(23)**, 3843–3854 (2009).
7. Kirby, D. P., Buckley, M., Promise, E., Trauger, S. A. & Rose Holdcraft, T. Identification of collagen-based materials in cultural heritage. *Analyst* **138(17)**, 4849–4858 (2013).
8. Welker, F. et al. Palaeoproteomic evidence identifies archaic hominins associated with the Châtelperronian at the Grotte du Renne. *Proc. Natl. Acad. Sci. U.S.A.* **113(40)**, 11162–11167 (2016).
9. Reimer, P. et al. The IntCal20 Northern Hemisphere radiocarbon age calibration curve (0–55 cal kBP). *Radiocarbon* **62(4)**, 725–757 (2020).
10. Bronk Ramsey, C. Bayesian analysis of radiocarbon dates. *Radiocarbon* **51(1)**, 337–360 (2009).
11. Rannamäe, E. et al. Maternal and paternal genetic diversity of ancient sheep in Estonia from the Bronze Age to the Post-Medieval Period, and comparison with other regions in Eurasia. *Anim. Genet.* **47(2)**, 208–218 (2016).
12. Rannamäe, E. et al. Three thousand years of continuity in the maternal lineages of ancient sheep in Estonia. *PLoS ONE* **11(10)**, e0163676; 10.1371/journal.pone.0163676 (2016).
13. Schroeder, O. et al. Endogenous retroviral insertions indicate a secondary introduction of domestic sheep lineages to the Caucasus and Central Asia between the Bronze and Iron Age. *Genes* **8(6)**, 165; 10.3390/genes8060165 (2017).
14. Arnaud, F. et al. A paradigm for virus–host coevolution: sequential counter-adaptations between endogenous and exogenous retroviruses. *PLoS Pathog.* **3(11)**, e170; 10.1371/journal.ppat.0030170 (2007).
15. Chessa, B. et al. Revealing the history of sheep domestication using retrovirus integrations. *Science* **324(5926)**, 532–536 (2009).
16. Arnaud, F., Varela, M., Spencer, T. E. & Palmarini, M. Coevolution of endogenous betaretroviruses of sheep and their host. *Cell. Mol. Life Sci.* **65(21)**, 3422–3432 (2008).
17. Bowles, D., Carson, A. & Isaac, P. Genetic distinctiveness of the Herdwick sheep breed and two other locally adapted hill breeds of the UK. *PLoS ONE* **9(1)**, e87823; 10.1371/journal.pone.0087823 (2014).

18. *Mason's World Encyclopedia of Livestock Breeds and Breeding* (ed. Porter, V., Alderson, L., Hall, S. J. G. & Sponenberg, D. P.) Volume 2, 6th edition (CAB International, 2016).
19. Grigaliūnaitė, I. et al. Microsatellite variation in the Baltic sheep breeds. *Veterinarija ir zootechnika* **21(43)**, 66–73 (2003).
20. Tapio, I. et al. Unfolding of population structure in Baltic sheep breeds using microsatellite analysis. *Heredity* **94(4)**, 448–456 (2005).
21. Dýrmundsson, Ó. R & Niżnikowski, R. North European short-tailed breeds of sheep: a review. *Animal* **4(8)**, 1275–1282 (2010).
22. Geoportal. Republic of Estonia Land Board https://geoportaal.maaamet.ee/index.php?lang_id=1&page_id=119 (2020).
23. Adobe Illustrator CS5 https://adobe.com (2010).
24. Palmarini, M. et al. Molecular cloning and functional analysis of three type D endogenous retroviruses of sheep reveal a different cell tropism from that of the highly related exogenous jaagsiekte sheep retrovirus. *J. Virol.* **74(17)**, 8065–8076 (2000).
